# *Citrus limon* Wastes from Part of the Eastern Cape Province in South Africa: Medicinal, Sustainable Agricultural, and Bio-Resource Potential

**DOI:** 10.3390/molecules29071675

**Published:** 2024-04-08

**Authors:** Phumelele Nodola, Gugulethu M. Miya, Vuyokazi Mazwi, Ayodeji O. Oriola, Opeoluwa O. Oyedeji, Yiseyon S. Hosu, Simon K. Kuria, Adebola O. Oyedeji

**Affiliations:** 1 Department of Chemical and Physical Sciences, Walter Sisulu University, Mthatha 5117, South Africa; jokweni.phumee@gmail.com (P.N.); gmiya@wsu.ac.za (G.M.M.); vmazwi@wsu.ac.za (V.M.); aoriola@wsu.ac.za (A.O.O.); 2 Department of Chemistry, University of Fort Hare, Alice 5700, South Africa; ooyedeji@ufh.ac.za; 3 Department of Business Management and Economics, Walter Sisulu University, Mthatha 5117, South Africa; yhosu@wsu.ac.za; 4 Department of Biological and Environmental Sciences, Walter Sisulu University, Mthatha 5117, South Africa; kkuria@wsu.ac.za

**Keywords:** agricultural and socio-economic benefits, antioxidant, *Citrus limon*, essential oils, insecticidal

## Abstract

The fruits of *Citrus limon* are often purchased for their vitamin C-rich juice, while the fruit peel and the tree leaves are discarded as wastes. This study obtained the chemical profiles of the essential oils (EOs) of *C. limon* wastes (the peel and leaves), evaluated their medicinal value as antioxidants, their potential for sustainable use in agriculture as an insecticide for post-harvest preservation of grains, and their potential as a bioresource in livestock feed formulations. The EOs were isolated from *C. limon* leaves and peel using a hydro-distillation method on a Clevenger apparatus. The oil constituents were identified using the gas chromatography-mass spectrometry (GC-MS) hyphenated technique. The oils were evaluated for their in vitro antioxidant activity using 2,2-diphenyl-1-picrylhydrazyl (DPPH) and ferric-reducing antioxidant power methods. An insecticidal study was conducted using contact toxicity, fumigation, and repellence bioassay methods against *Sitophilus zeamais* (maize weevils). Finally, the predicted income from using lemon peel as an alternative or substitute ingredient for maize in livestock feed formulations was obtained through a conventional simulation method. Chemically, limonene was found to be present in all the EOs analyzed (12–52%), while α-pinene was only found in the fresh leaf and peel oils (13.3% and 10.6%). Caryophyllene oxide was identified as the major component of the dried leaf oil (17.7%). At 20 µg m, the dry peel oil exhibited the highest inhibitory activity (52.41 ± 0.26%) against the DPPH radical, which was comparable to L-ascorbic acid (a standard antioxidant) at 54.25 ± 3.55%. The insecticidal study revealed that the dry peel oil is a better insect repellent (73.33 ± 6.95% at 10 µL) and fumigant (LC_50_ = 0.17 µL g^−1^ after 48 h) natural agent compared to the peel oil. Conversely, the dry peel oil showed a better contact activity (LC_50_ = 1.69 µL g^−1^) against the maize weevils compared to the dry leaf oil. The simulation study showed the cost of using dry lemon peel as an alternative to maize in livestock feed formulation to be ZAR 2.8 billion, compared against the higher cost of feed formulation with maize, which currently stands at ZAR 24.9 billion. This study has shown that *C. limon* wastes (the peel and leaves) contain EOs with unique chemical profiles, valuable medicinal properties as free radical scavengers, and considerable insecticidal properties for agricultural use in post-harvest grain preservation, presenting a cost-effective and promising bioresource for livestock feed production.

## 1. Introduction

Citrus fruit is one of the most important fruit crops in the world, with a global production of approximately 102 million tons yearly [1,2]. It is well known for its dietary, medicinal, and agricultural benefits, to mention a few, because of its valuable source of bioactive compounds [3]. Unfortunately, the industrial processing of citrus fruits leaves a huge burden of bioaccumulated wastes, as citrus by-products (peels and leaves) have been reported to account for one-fifth of the total industrial wastes generated globally, which translate to about 120 million tons of citrus-generated wastes annually [4,5]. Thus, a significant amount of these waste products is released into the environment, causing serious environmental hazards. Nonetheless, these by-products are known to contain unique phytochemicals, making them medicinally beneficial to human health, agriculturally useful, and economically valuable [6,7]. Amongst the popular citrus wastes are those produced from *Citrus limon*, commonly called the “Lisbon lemon”, which comes after *C. sinensis* (the orange) and *C. reticulata* (the mandarin) [8]. 

*C. limon* (L.) Burm. f. (family Rutaceae) grows as a small thorny tree, reaching a height of about 20 ft, with alternately arranged dark-green leaves [9]. It is an important medicinal plant cultivated mainly for its juice and spice [2]. Like many citrus fruits, *C. limon* is known to contain phytochemicals, ranging from fatty acids to phenolics and flavonoids (catechin, rutin, hesperidin, and naringin), pectin, and EO compounds such as limonene, α-terpineol, γ-terpinene, 4-terpineol, α-phellandrene, β-myrcene, α-pinene, β-linalool, and α-selinene [10,11,12,13,14,15].The fruit peel and leaves from Lisbon lemons have been reported for several biological properties, including antioxidant, antibacterial, antifungal, anticancer, and antiviral activities [16]. Lemon peel oil is beneficial to the skin when consumed orally or applied externally for it rejuvenates the skin, keeps it shining, protects it from infections, and reduces body odor [17]. *C. limon* as a fruit is also said to be rich in potassium, which makes it very effective in the removal of toxic substances and precipitates deposited in the kidneys and urinary bladder, and its disinfectant properties help cure infections in the urinary system [9,18]. The antioxidant and antimicrobial activities of lemon peel extracts used for skin diseases by the Xhosa tribe of the Amathole District, Eastern Cape, South Africa have been reported [19]. 

Lemon wastes have also been described as a valuable bioresource for agricultural production. For instance, lemon peel EO and its nano-formulation have proven to be effective for the control of black cutworms, *Agrotis ipsilon* (Hufnagel) (Lepidoptera: Noctuidae), a destructive pest that economically cause an extensive loss of many crops including maize, cotton, wheat, and many vegetables [20]. In another study, the EOs from four citrus peel (*C. limetta*, *C. limon, C. reticulata*, and *C. aurantifolia*) wastes from India showed significant contact, fumigant, and repellent activities against two key stored-grain pests, *Tribolium castaneum* (Herbst) and *Callosobruchus chinensis* (L.), with *C. limon* having the highest contact and fumigant toxicity and repellence [21]. Furthermore, residues from lemon juice extraction are potentially an excellent source of dietary fiber because of their rich pectin content [22]. In a study on cases of dietary fiber from fruit, lemon varieties were reported to have the highest amount of total dietary fibers, making lemon peel a potential ingredient in livestock feeds [23]. Thus, it is expected that the application of *C. limon* wastes as an important bioresource in post-harvest grain preservation and livestock feed formulations may help to achieve both human and animal food security, especially in countries where these raw materials are largely produced.

South Africa is among the world’s major citrus-producing countries, recording about 2.1 million tons of citrus production in the year 2011/2012 [24,25]. The Eastern Cape province in South Africa produces about 46% of the country’s lemons and this region is forecast to be less affected by the current drought conditions compared to the other eight provinces [26,27]. Hence, lemon production is projected to increase, and so is lemon waste accumulation. This, in turn, provides an opportunity to repurpose lemon wastes for medicinal, agricultural, and socio-economic use. Therefore, this study investigated the waste products (peel and leaves) of *C. limon* grown in part of the Eastern Cape province in South Africa for their EO constituents, and determined their antioxidant, insecticidal, and bioresource potential for medicinal, sustainable agricultural, and socio-economic use.

## 2. Results

### 2.1. Physicochemical Properties of C. limon Oils

Physicochemical parameters such as the color, smell, percentage yield, and specific gravity are an important tool for EO characterization. Therefore, the afforded EOs were characterized by their smell, color, and percentage yield, ranging from 0.39% to 4.75%, as shown in Table 1. The study revealed that the oil yields (*w*/*w*) from the dried material doubled that of the fresh material, while the highest oil yield was observed in the dry peel. The color of the fresh *C. limon* fruit peel and leaf EOs were observed to be colorless, while the dried fruit peel and leaf oils were pale yellow (Table 1).

### 2.2. GC/MS Analysis of EOs of C. limon Leaves and Peel

Gas chromatography–mass spectrometry is a powerful hyphenated technique for the easy separation of EOs and their chemical detection [28]. It was used in this study for the comprehensive detection and quantitation of compounds contained in the lemon peel and leaf oils. Based on the GC/MS result, twenty-seven (27) compounds were identified on the chromatogram of the fresh fruit peel EO from *C. limon*, accounting for 81.9% of the total oil constituents, with β-pinene (10.6%), limonene (52.5%), γ-terpinene (8.8%), and geranial (5.7%) as the prominent compounds. The dried fruit peel had a total of 51 identified compounds (86.3%), with limonene (36.0%), alloocimene (5.6%) and L-carvone (5.9%) being the major compounds (Table 2). The fresh leaf oil had 39 identified compounds (96.4%), with limonene (31.0%), β-pinene (13.3%), citral (10.3%), and geranial (13.2%) being the major compounds. Twenty-one compounds were identified in the dried leaf EO, which gave 82.4% of the total oil composition. However, linalool (8.6%), limonene (12.0%), caryophyllene oxide (17.7%), and limonene glycol (5.2%) were the most prominent compounds in this oil profile. It was observed that three of the EOs—the fresh fruit peel, dried fruit peel, and fresh leaf EOs—had limonene as the dominant compound. The sesquiterpenoid caryophyllene oxide was identified as the prominent compound in the dried leaf oil.

### 2.3. Antioxidant Activity of C. limon Essential Oils

#### 2.3.1. Ferric-Reducing Power

**Figure 1:**
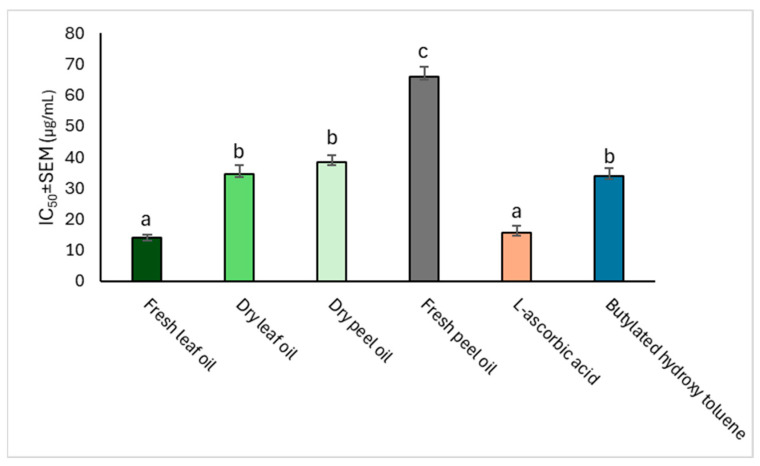
Antioxidant activity ranking of *C. limon* essential oils based on their ferric-reducing antioxidant power (IC_50_ values). Ranking: fresh leaf oil = L-ascorbic acid > dry peel oil = butylated hydroxy toluene = dry leaf oil > fresh peel oil. Different letters between the bars indicate that the groups are significantly different at *p* < 0.05, while bars with the same letters indicate no significant differences (*p* > 0.05).

The antioxidant potential of the *C. limon* oils was measured spectrophotometrically in terms of their electron-donating ability to reduce ferric ions (Fe^3+^) to ferrous ions (Fe^2+^). The results in Table 3 show that the fresh leaf oil exhibited the highest ferric-reducing power (≈50%) at the lowest concentration (20 µg mL^−1^). This activity was comparable to those of L-ascorbic acid (ASC) and butylated hydroxy toluene (BHT), the positive controls used. Furthermore, Figure 1 reveals the ferric-reducing power ranking for the tested oils, showing that the fresh leaf oil gave the best activity based on its least IC_50_ of 14.10 ± 0.97 µg mL^−1^, while this activity was comparable (*p* > 0.05) to that of L-ascorbic acid (IC_50_ of 15.50 ± 1.10 µg mL^−1^).

#### 2.3.2. DPPH Radical-Scavenging Potential

DPPH radical scavenging is a standard, very fast, and practical technique widely used in antioxidant activity studies. It is an ideal method for the assessment of herbal extracts, including EOs and phenolic compounds [29]. Each of the EOs exhibited a higher percentage of DPPH-scavenging activity than BHT at 160–40 µg mL^−1^. The scavenging effect of all tested oils distinctively showed a concentration-dependent increase. At the highest concentration of 160 µg mL^−1^, L-ascorbic acid showed a high scavenging effect of 92.81 ± 0.95%, followed by the dried peel and leaf oils (87.66 ± 0.39 and 84.01 ± 0.45%). However, BHT had the lowest DPPH-scavenging effect (82.60 ± 1.56%) (Table 4). In terms of the ability of the EOs to cause a 50% inhibition of the DPPH free radical, which represents the IC_50_ (Table 4), the dry peel oil demonstrated the best activity (8.79 ± 1.30 µg mL^−1^), closely followed by the dry leaf oil (12.59 ± 1.48 µg mL^−1^). Both EOs were observed to be significantly (*p* < 0.05) better than BHT, one of the standards used.

### 2.4. Insecticidal Properties of C. limon Oils against Sitophilus zeamais

Insect repellency, fumigant toxicity, and contact toxicity assays are conventional methods for evaluating the biological efficacy and effectiveness of substances, including EOs and other natural extracts, against insects (pests). These biological indices are an integral part of the profile and the mode of application for selecting potent insecticidal and/or pesticidal agents [30]; hence, they were adopted in this study. 

#### 2.4.1. Repellent Activity

The dry leaf and peel oils showed promising antioxidant properties to warrant further insecticidal investigation for their possible use in the post-harvest preservation of maize. The repellent effects of the dried leaf and peel *C. limon* EOs against *S. zeamais* are presented in Table 5. All studied EOs were repulsive at all the test concentrations against *S. zeamais*, but the highest repellent effect was observed at 40 µL. Generally, at this concentration, *C. limon* oils showed strong activity with a repellent percentage range of 78–82%. However, the dry leaf oil demonstrated a higher repellent activity (82.67 ± 4.73%) at a 30 µL concentration.

#### 2.4.2. Fumigant Toxicity

The fumigant toxicity of *C. limon* EOs was subjected to a probit analysis to calculate the insecticidal potential of the oils against *S. zeamais*. The LC_50_ values and the corresponding confidence intervals of the oils are shown in Table 6. The dry leaf oil exhibited better fumigant toxicity against *S. zeamais* (0.17 µL mL^−1^) after 48 h of application, as compared to the dry peel EO (0.35 µL mL^−1^) at the same time.

#### 2.4.3. Contact Toxicity

The LC_50_ values and the corresponding confidence intervals were determined for the South African *C. limon* EOs through the introduction of *S. zeamais* on treated maize. The contact toxicity effects of the *C. limon* dry peel and leaf EOs against *S. zeamais* within the 24–72 h time interval is shown in Table 7. Both oils showed contact toxicity against *S. zeamais*, with LC_50_ values ranging between 1.39 µL g^−1^ and 4.09 µL g^−1^ at 24–72 h (Table 7).

### 2.5. Agricultural and Socio-Economic Benefits of C. limon Peel

Simulations are a valuable agricultural tool for testing real-world situations and processes without actually putting them into action. As such, working with simulations can save time and resources since they help to identify where changes can be made before processes are implemented [31]. Therefore, in this study, a simulation of the benefits of using lemon peel was carried done and the results are presented in Table 8. According to the GAIN report in [26], South Africa produced 340,000 MT of lemons of 2016. Of these, the country exported 240,000 MT, leaving 100,000 MT for both internal consumption and wastage. Given that the dehydrated peel from one lemon weighs 9.19 g on average, we can convert the metric tons of the available lemon peel waste to be used as a major substitute for energy-producing ingredients in livestock feed formulations within South Africa. The results in Table 8 indicate that, at the levels of production and local utilization of lemons seen in 2023, one-tenth of the maize used as an ingredient in the production of livestock feed can be substituted with lemon peel waste generated within South Africa. With the price of a ton of maize selling at 5061 South African rands (ZAR 5061; January 2024 price), up to ZAR 3,856,482,000.00 could be realized from lemon peel waste generated within the country, using it as alternative ingredient for livestock feed production in place of the maize used currently.

## 3. Discussion

It was observed that the fruit peel gave higher EO (*w*/*w*) yields than the leaves from both fresh and dried materials. Based on the results presented in Table 2, the dry peel oil contains a higher number of EO constituents (51) than the fresh peel oil (26). Perhaps new EO compounds might have been formed during the drying process, as a previous report has shown that the constituents of lemon peel oil increase with drying [32]. Conversely, another report has shown a marked reduction in the total qualities and quantities of EOs in the cases of sweet basil, marjoram, and oregano at ambient temperatures [33]. Therefore, it may be difficult to predict the release or retention of volatile components of EOs because of the interplay of factors such as the kind of spice used, the nature of the chemotypes in the spice, and the drying method and its duration, as well as other environmental factors and phenological parameters [34,35]. Table 2 also shows limonene as a major citrus constituent present in all of the afforded oils at high percentages of the overall composition, ranging from 12.0 to 52.5%. This result agrees with those reported in the literature, where lemon peel cultivated in Brazil contained 70.8% limonene, while Indian and Polish oils showed 53.6% and 48.3%, respectively [10,12,13]. Limonene is well known as an antiviral agent and for its ability to detoxify the liver and prevent tumor development [36]. 

The dominance of the oxygenated sesquiterpene caryophyllene oxide at a high concentration (17.7%) in the dried leaf oil is worth noting as it has not been reported before in the literature. The dominance of sesquiterpenoids in the oil could partly be responsible for the higher mass and percentage yield of the EOs obtained from the dry peel and leaves compared to their fresh leaf and peel counterparts. A similar scenario was reported in the EO-rich plant *Felicia muricata*, in which the dry leaf oil with a higher composition of sesquiterpenes showed a higher oil yield than its monoterpene-rich fresh leaf oil counterpart [35]. Biologically, caryophyllene oxide is known for its antioxidant, anti-inflammatory, neuroprotective, and anticancer properties [37,38,39]. Thus, the presence of limonene, caryophyllene oxide, and other bioactive EO constituents in *C. limon* may further confer considerable medicinal properties on the Lisbon lemon plant grown in South Africa.

**Figure 2:**
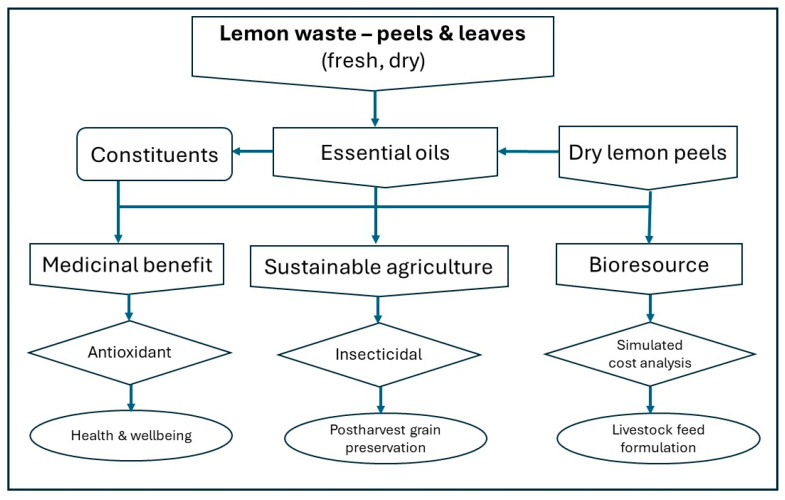
Study flow chart.

The results of the antioxidant activity assay of the EOs of *C. limon* in Table 3 and Table 4, and Figure 1 and Figure 2, show the considerable antioxidant properties of the oils, and, by implication, the medicinal, agricultural, and socio-economic prospects of these citrus wastes (Lisbon lemon peel and leaves). Interestingly, the EOs from the fresh leaf and dry peel oils exhibited much higher ferric-reducing antioxidant power than the others, which is suggestive of their ability to effectively engage in single-electron transfer by releasing an electron to free radicals and converting them to anions. This reaction prevents further elongation of the free radical chain, reducing its ability to continuously produce free radicals, and preventing oxidative damage to the DNA and lipids [40]. It was also revealed through this study that the dry peel and leaf oils exhibited better DPPH radical-scavenging activities compared to the BHT standard and even those oils obtained from the fresh lemon. This implies that the dry lemon oils can counteract the continuous production (accumulation) of free radicals in the biological system through their effective proton-donating ability [41]. Our study findings corroborate a recent report by Himed et al. [42], in which the peel EO of the Eureka variety of *C. limon* in Algeria showed a DPPH radical-scavenging IC_50_ activity of 660 µg mL^−1^, with limonene (67.1%), α-pinene (11.0%), and α-terpinene (8.0%) being identified as the oil’s major components [42]. This warrants further studies on the insecticidal potential of these oils for possible use in the post-harvest preservation of maize.

The potential use of *C. limon* wastes (peel and leaves) in maize preservation from the obtained results (Table 5, Table 6 and Table 7) indicates that both the leaf and the peel oils have good repellent, contact, and fumigant activities. The considerable insecticidal properties of the oils could be linked to their monoterpene contents, namely limonene, α-pinene, and γ-terpinene, which altogether constitute >70% of the oils. A 1.0% limonene spray has been reported to be superior to synthetic insecticidal soaps, achieving 99% control of white flies, 99% control of mealybugs, and 93% control of root mealybug insects, while causing no damage to ornamentals with thick, waxy leaves, such as cycads, orchids, and palms [43].

Based on the aforementioned antioxidant and insecticidal properties of *C. limon* EOs, the plant was further investigated for the possible agricultural and socio-economic benefits that can be derived from its by-products. In this study, lemon peel was selected and a simulation was used to predict its agricultural and socio-economic benefits. The selection of lemon peel is justified by empirical evidence indicating the non-biodegradable nature of citrus peels and a propensity for this waste material to cause environmental hazards, while on the other hand, they also have been known to contain high energy/dietary fiber [44]. Generally, because the processing technology is simple, both smallholder and commercial farmers can convert this peel waste into useful agricultural products that will reduce the current land degradation in South Africa because of overstocked livestock. It is expected that an finding alternative to maize as a major ingredient for livestock feed production in South Africa will affect the demand and supply of maize, and subsequently cause a decline in the price of maize in South Africa. The animal feed industry primarily uses yellow maize for the purpose of manufacturing animal feed. Approximately 60% of the total maize produced in South Africa is used for food consumption, industrial (other than feed), and seed purposes, while the rest is used to produce animal feed. The annual maize production is about 10.5 million tons, of which about 4 million tons are used in the starch industry, 6.65 million tons are used in animal feeds, and 6.1 million tons are used for human consumption and seed production. Maize is a major ingredient in feed milling, constituting up to one half of the total volume of ingredients used [26]. Replacing the maize in animal feeds with alternative ingredients is expected to cushion the impact of demand-induced inflation on the poor, because maize is a staple food in South Africa. It is also important to indicate that up to ZAR 22 billion could be saved from the national budget if lemon peel is harnessed as an alternative to or a substitute ingredient for maize in livestock feed formulations in South Africa (Table 8). Thus, lemon peel wastes can immensely contribute to mitigating the current budget deficit of the country, delivering tangible socio-economic benefits for national growth.

We recommend that more work be conducted on South African *Citrus limon* wastes vis-à-vis other species of citrus, since the current study has shown the unique essential oil contents of the plant’s peel and leaves, their antioxidant nature, and their insecticidal properties for possible use in the post-harvest preservation of maize, as well as the related sustainable agricultural and socio-economic benefits for South Africa.

## 4. Materials and Methods

### 4.1. Plant Material

*Citrus limon* (Lisbon lemon) leaves and fruits (1200 g each) were harvested from selected sites of the Addo River Bend commercial farm (33°25′33.7″ S 25°45′11.9″ E) in the Eastern Cape province, South Africa. The plant was identified at Kie Herbarium, Walter Sisulu University, Eastern Cape, South Africa, and a voucher specimen (Phume 001) was kept at the Kie Herbarium for future reference.

### 4.2. Extraction of EOs from C. limon Leaves and Peel

The fresh and dry leaves and peel of *C. limon* were extracted for their EOs through a hydro-distillation method using Clevenger apparatus [45]. To achieve this, 600 g each of fresh and dried lemon leaves and peel was introduced into a 5 L round-bottomed flask, and 3.0 L of distilled water was added as an extraction solvent for each plant material. The flask was placed on the heating mantle at a temperature of 100 °C and was connected to a condenser until the mixture boiled. Then, the temperature was lowered to 70 °C and run for 4 h to obtain the EOs [46]. The oils were stored in air-tight amber bottles and kept in a refrigerator until needed for further analysis.

### 4.3. GC and GC/MS Analysis of EOs

GC analysis was carried out on a Bruker gas chromatograph with an FID detector and a BP-1 capillary column (30 m × 0.25 mm; film thickness 0.25 µm). The operating conditions were as follows: the carrier gas was helium with a flow rate ranging from 6–10 mL/min at 1 mL/min; the column temperature ranged from 50 to 280 °C at 5 °C/min; the injector and detector temperature was 280 °C; the volume of oil injected was 0.1 µL, with a split ratio of 1:50. The GC/MS analysis was performed on a Hewlett Packard 6890 MS selective detector coupled with a Hewlett Packard 6890 gas chromatograph equipped with a cross-linked 5% PHME siloxane HP-5MS capillary column (30 m × 0.25 mm; film thickness 0.25 µm) and operating under the same conditions as described above. The MS operating parameters were as follows: ionization potential 70 eV, ionization current 2A, ion source temperature 200 °C, and the column oven temperature was programmed from 50 to 325 v °C. The *m*/*z* was set at 40–600 and resolution 1000. The percentage of the samples and quantification of components were computed from the GC peak areas. Identification of the EO constituents was based on the literature and fragmentation patterns of compounds in the system database.

### 4.4. Antioxidant Analysis of C. limon EOs

#### 4.4.1. Ferric-Reducing Antioxidant Power (FRAP) Assay

The ferric-reducing antioxidant power of the EOs was evaluated according to the method of Okeleye et al. [46], with some modification. Exactly 100 µL of the oil was added to a mixture containing 250 µL of phosphate buffer (0.2 M; pH 6.6) and 250 µL of potassium ferrocyanide [K_3_Fe(CN)_6_] (1% *w*/*v*). The standard compounds used were L(+)-ascorbic acid and 2,6-di-tert-butyl-4-methyl phenol, also known as butylated hydroxy toluene (BHT). The resulting mixture was incubated at 50 °C for 20 min, followed by the addition of 250 µL of CCl_3_COOH (10% *w*/*v*), and then centrifuged at 3000× *g* rpm for 10 min. The upper layer of the solution (250 µL) was mixed with 250 µL of distilled water and 50 µL of FeCl_3_ (0.1%, *w*/*v*). The experiment was conducted in triplicates, and the absorbance was measured at 700 nm on a microplate reader (Helios Epsilon Thermo Spectronic, Waltham, MA, USA) against a blank sample of only phosphate buffer. The ferric-reducing antioxidant power of the essential oils was determined using UV–visible absorbencies at 700 nm and it was measured according to the increase in absorbance, whereby an oil had a higher reducing power if it had a higher absorbance. An important mechanism of phenolic antioxidant action is Fe^3+^ reduction, which is often used as an indicator of electron-donating activity. The antioxidant potential of the oils was estimated from their ability to reduce Fe^3+^ to Fe^2+^ with an observable change in coloration from yellow to green after the addition of ferrous chloride. The activity was expressed as percentage Fe^2+^-reducing ability, according to Equation (1):

(1) $$\%\;Ferric\;reducing\;antioxidant\;power=\frac{Ac-As}{Ac}\times 100$$

where *Ac* = absorbance of the negative control, *As* = absorbance of the test sample. The concentration that caused a 50% ferric reduction represented the IC_50_ value.

#### 4.4.2. DPPH Radical Scavenging Assay

The total antioxidant capacity of each oil was measured using the 2,2-diphenyl-1-picrylhydrazyl (DPPH) radical as previously described by Shen et al. [47], with some modifications. A solution of 0.135 mM DPPH in methanol was prepared and 180 µL of this solution was mixed with 180 µL of the lemon oils dissolved in 40 µL of Tween 20. DPPH in methanol was used as a negative control. The combination was thoroughly mixed and left in the dark at room temperature for 30 min before the absorbance was measured at 517 nm on the microplate reader (Helios Epsilon Thermo Spectronic, Waltham, MA, USA). L(+)-ascorbic acid and DBPC*BHT were used as the standard drugs. The activity was expressed as percentage *DPPH radical-scavenging activity*, based on Equation (2):

(2) $$\%\;DPPH\;radical\text{-}scavenging\;activity=\frac{Ac-As}{Ac}\times 100$$

where *Ac* = absorbance of the negative control, *As* = absorbance of the test sample. The concentration that caused a 50% inhibition of the DPPH radical represented the IC_50_.

### 4.5. Insecticidal Study on C. limon Leaf and Peel EOs

#### 4.5.1. Test Insects

The study was approved by the Faculty Research Ethics Committee, with ethical approval reference WSU/FNS-GREC-2018/01-12/G11. Maize weevils (*Sitophilus zeamais* Motschulsky) were collected from household storages in the Mthatha township, Eastern Cape province, South Africa (31°35′20.1″ S 28°47′3.9″ E) in March 2019. The weevils were cultured at the Entomology Laboratory in the Department of Biological Sciences, Walter Sisulu University, South Africa, following the standard procedure described by Yang et al. [48], with slight modification. At the laboratory, the weevil colony was maintained in a 1.0 L container layered with 300 g of maize grains (*Zea mays*). The weevils were kept at room temperature (≈25 °C) under a light-to-dark photoperiod of 14:10 h. Twenty-one-day-old active and unsexed adult weevils were selected from the colony pool and used for the experiments.

#### 4.5.2. Repellent Assay

The repellent effects of dried *C. limon* EOs against *S. zeamais* were studied according to Tapondjou et al. [49], with slight modifications. The test area consisted of a 9 cm Whatman No.1 filter paper cut into two equal halves. Different oil concentrations were prepared by diluting 10, 20, 30, and 40 µL of the oil with 1 mL of *n*-hexane and these corresponded to concentrations of 0.31, 0.47, 0.94, and 1.26 µL of oil/cm^2^ on one half of the filter paper, respectively. The other half was treated with 0.5 mL of *n*-hexane alone, to serve as a control. Both the EO-treated and *n*-hexane-treated filter papers were air-dried for 10 min to evaporate the solvent. With the aid of a transparent tape, both halves were later joined into full discs and placed in 9 cm diameter glass Petri dishes. A total of ten maize weevils were released at the center of the re-joined filter paper disc and then a cover was placed over the Petri dish. Each treatment was replicated 3 times using a factorial design. The number of individual insects present in the control (*Nc*) and treated (*Nt*) areas of the filter paper were recorded 1, 2, 4, 6, and 24 h after release. The percentage repellency (*PR*) was calculated as indicated in Equation (3):

(3) $$PR=\frac{Nc-Nt}{Nc+Nt}\times 100$$

Concentration was calculated by dividing the oil quantity (µL) by the surface area of half the filter paper (31.81 cm²). Results were presented as the mean percentage repellency ± the standard error of the mean (SEM).

#### 4.5.3. Fumigant Assay

The fumigant assay of the *C. limon* dried peel and leaf EOs was performed as described in Peebles et al. [50]. The fumigation chambers consisted of 350 mL glass jars with screw-on lids. Solutions of 0, 8, 16, 32, and 40 µL of the oils were diluted with 1 mL of *n*-hexane to correspond to concentrations of 0 (control), 0.02, 0.05, 0.09, and 0.11 µL mL^−1^, calculated by dividing the oil concentration by the volume of the fumigant chamber, and these converted concentrations were used for the probit analysis. A portion of 1 mL of each essential oil concentration was separately applied to 7 mm diameter discs of Whatman No.1 filter paper, air-dried for 10 min, and placed at the bottom of the jars. A total of ten *S. zeamais* insects were placed on muslin cloths measuring 21 × 29 mm each with 20 g dried whole-maize grains. The cloths were tied closed with rubber bands and hung at the center of the jars, which were then sealed with air-tight lids. *S. zeamais* insects were considered dead if they showed no movement when probed with sharp objects. Three replicates of the treatments and untreated controls were laid out in a factorial experimental design. Fumigation was carried out for 24 h, after which the insects were transferred from the fumigation chambers onto clean dried maize, and mortality was checked daily after 24, 48, 72, and 96 h.

#### 4.5.4. Contact Toxicity Test

The contact toxicity of the *C. limon* dry peel and leaf essential oils against *S. zeamais* was investigated according to the modified method of Peebles et al. [50]. Maize grains were treated with concentrations of 0, 50, 100, 200, and 300 µL of the essential oil in 1 mL of *n*-hexane. The different concentrations of the oil were mixed with 40 g of the maize grains, respectively, resulting in the following concentrations: 0, 1.25, 2.50, 5.00, and 7.50 µL/g, respectively. These were thoroughly stirred to allow for homogeneity of the oil on the treated grains. The treated samples were air-dried for 1 h to remove the solvent. Thereafter, the maize grains were infested with twenty *S. zeamais* individuals per jar (300 mL), and each jar was covered with a cotton mesh held in place by cover rims. *S. zeamais* were considered dead if they showed no movement when probed with sharp objects. Three replicates of the treatments and untreated controls were laid out in a factorial experimental design. *S. zeamais* mortality was checked daily after 24, 48, and 72 h. The log-converted concentrations were used for the probit analysis.

### 4.6. Statistical Analysis

The mean mortality against all tested Eos was calculated according to an analysis of variance (ANOVA) using a factorial design. The ANOVA results that revealed significant differences were then subjected to post hoc analysis through the Duncan test using a statistical program. Values with *p* < 0.05 were considered significantly different. The concentrations and mortality data for both the fumigant and contact toxicity assays were subjected to probit analysis using the SPSS 25.0 statistical program to calculate the LC_50_ values. The lethal concentration was significantly different if the 95% confidence limit did not overlap. In a probit analysis, higher *p*-values indicate that the model fits the data well, whereas lower *p*-values indicate that the predicted probabilities from the model differ significantly from the observed probabilities in the data.

### 4.7. Simulation of Sustainable Agricultural and Socio-Economic Benefits of Lemon Peel

This study was conducted according to the GAIN report in [26]. Lemon peel was extracted from five lemon fruits. The wet weights of the peel were taken. Thereafter, the peel was dehydrated (sun-dried to ensure a crispy texture) and the dry weight measurements were taken. The average weights of the wet and dried extracted lemon peel were taken to determine what the dry matter of the peel from a lemon fruit weighed. The average dry matter weight of the lemon was used to simulate the agricultural and socio-economic benefits, since lemon peel has been found to be of high energy/dietary fiber (even after sun-drying or drying in a silo). The simulation was carried out to calculate the income from using lemon peel as an alternative or substitute ingredient for maize in livestock feed formulations.

A flow chart of the study methodology illustrating the three studied aspects and the parameters studied is presented in Figure 2.

## 5. Conclusions

This study has shown that the leaf and peel wastes of *C. limon* cultivated in part of the Eastern Cape province in South Africa contain EOs with unique chemical profiles. The oils showed marked variations in the quality and quantity of their constituents, with the dry peel giving the highest oil yield and the highest number of EO constituents. Limonene, α-pinene, and γ-terpinene are among the major monoterpenes in the oils, while caryophyllene oxide is reported for the first time as a major sesquiterpenoid in the dry leaf oil. Furthermore, the oils showed considerable free radical-scavenging activity and insecticidal properties (repellency, fumigant toxicity, and contact toxicity), thus highlighting their potential for agricultural use as biopesticides for post-harvest grain preservation. It has also been revealed through this study that dried lemon peel can be a cost-effective alternative bioresource for maize in livestock feed formulations. 

## Figures and Tables

**Table 1:** Physiochemical analysis of *C. limon* fruit peel and leaf EOs.

*Citrus limon* EO	EO Smell	EO Color	Starting Material (g)	Mass of Oil (g)	% (*w*/*w*)
Fresh leaf	Sharp pungent citric smell	Colorless	600.00	2.59	0.39
Dried leaf	Herbaceous smell	Yellowish	600.00	4.14	0.69
Fresh peel	Sweet lemonade smell	Colorless	600.00	4.98	0.83
Dried peel	Sharp lemon smell	Light yellowish	600.00	9.72	1.62

**Table 2:** Chemical composition of EOs from *C. limon* leaves and fruit peel.

Compound	KI Value	Percentage Composition (%)			
		*C. limon* (Peel)		*C. limon* (Leaf)	
		Fresh	Dry	Fresh	Dry
Thujene	932	0.3	-	-	-
α-Pinene	934	1.5	0.2	1.1	-
Sabinene	957	0.3	0.3	-	-
δ-3-Carene	974	-	-	1.0	-
β-Pinene	980	10.6	2.2	13.3	-
Myrcene	991	1.4	-	1.4	-
6-Methyl-5-hepten-2-one	989	-	0.3	2.7	1.8
Octanal	1019	0.1	0.5	-	-
1,8-Cineole	1022	-	0.2	-	-
Limonene	1034	52.5	36.0	31.0	12.0
Limonene glycol	1035	-	-	-	5.2
1,8 Cineole	1036	-	-	1.2	2.1
Trans-β-ocimene	1047	0.1	0.3	2.3	-
Linalool oxide	1056	-	-	-	3.8
γ-Terpinene	1066	8.8	-	0.5	8.6
Cyclo-octane	1071	-	0.1	-	-
Epoxylinalool	1079	-	0.7	-	-
α-Terpinolene	1084	0.5	-	0.3	-
Trans-linalool oxide	1088	-	0.7	0.2	4.2
*p*-Cymene	1092	-	0.2	-	-
Linalool	1099	1.2	1.3	2.3	
Nonanal	1104	0.2	0.2	-	-
Fenchol	1117	-	0.1	-	-
Trans-*p*-2,8-menthadien-1-ol	1122	-	1.1	-	-
Cis-limonene oxide	1135	-	1.7	0.1	-
Trans-pinocarveol	1152	-	0.8	-	-
Citronellal	1155	-	0.2	0.3	-
Camphor	1159	-	0.2	-	-
*p*-Menth-1-en-9-al	1162	-	0.2	-	-
Pinocarvone	1163	-	0.8	-	-
Borneol	1165	-	0.3	-	-
1-Tert-butyl-3,3-dimethylcyclopropene	1181	-	1.3	-	-
Terpinen-4-ol	1182	1.1	1.1	1.0	1.5
α-Terpineol	1192	2.7	-	1.5	-
Epoxy-linalol	1198	-	-	-	1.1
Alloocimene	1207	-	5.6	-	-
2-Butenal, 3-methyl	1209	-	0.5	-	-
Citronellol	1211	-	0.7	-	1.8
Nerol	1228	1.7	-	0.7	2.3
Carveol	1233	0.3	-	-	1.0
Trans-(+)-carveol	1235	-	3.5	-	-
Exo-2-hydroxycineole	1238	-	0.5	-	-
Z-Citral	1240	4.3	0.7	10.3	-
Cis-carveol	1242	1.0	-	-	-
Geraniol	1245	1.3	4.1	1.3	1.3
S-Carvone	1249	0.3	-	0.2	1.3
Piperitone	1251	-	0.2	-	-
Cis-salvene	1254	-	0.1	-	-
Geranial	1258	5.7	-	13.2	4.1
Perillaldehyde	1270	-	0.3	-	-
Methyl geraniate	1323	-	-	0.2	-
Citronellyl acetate	1338	-	-	0.3	-
Farnesol	1345	-	0.3	-	-
α-Humulene	1443	-	-	0.1	-
Neryl acetate	1470	1.3	2.7	4.6	2.6
Spiro(5.6)dodecane	1496	-	-	-	1.8
D-Nerolidol	1539	-	0.2	-	-
1,10-Decanediol	1548	-	-	0.2	-
Geranyl acetate	1560	0.5	-	4.4	-
(+) Spathulenol	1579	-	-	-	3.9
β-Caryophyllene	1594	0.3	-	0.5	-
(-)-Humulene epoxide II	1603	-	-	-	1.3
Zingiberene	1611	0.6	0.2	-	-
Trans-sobrerol	1623	-	0.1	-	-
4-Methyl-5-vinyl thiazole	1673	-	0.2	-	-
1-(Dimethylamino)-3-borolene	1681	-	0.2	-	-
Geranylacetate,2,3-epoxy	1699	-	0.2	-	-
5-Methyl-tetradecane	1710	-	1.2	-	-
Valencene	1726	-	0.3	-	-
Dimethyl-2,6-octadienoic acid	1730	-	-	-	2.8
β-Bisabolene	1788	1.0	0.4	-	-
Caryophyllene oxide	1962	-	0.5	0.2	17.7
Isonicotinic acid	2088	-	0.1	-	-
3,5-Dimethyladamantan-1-ol	2102	-	0.3	-	-
(+,-)-(Z)-Dihydrofarnesal	2166	-	0.1	-	-
Trans-chrysanthenol	2174	-	0.2	-	-
Total % of compounds		81.9	86.3	96.4	82.4

Absent (-); kovat index (KI).

**Table 3:** Ferric-reducing antioxidant power of *C. limon* EOs.

Concentration (μg mL^−1^)	Mean Percentage Ferric-Reducing Power (%)					
	Fresh Leaf	Dry Leaf	Dry Peel	Fresh Peel	ASC	BHT
160	65.91 ± 1.21 ^b^	65.43 ± 1.19 ^b^	64.30 ± 1.07 ^b^	63.65 ± 0.29 ^b^	63.81 ± 0.33 ^b^	58.48 ± 0.97 ^a^
80	64.94 ± 0.43 ^c^	61.71 ± 1.26 ^b^	63.81 ± 0.54 ^bc^	63.33 ± 0.20 ^b^	63.33 ± 0.24 ^b^	56.06 ± 1.03 ^a^
40	62.19 ± 0.25 ^c^	53.63 ± 2.18 ^a^	58.64 ± 1.69 ^b^	57.51 ± 1.55 ^b^	57.51 ± 0.94 ^b^	52.99 ± 1.36 ^a^
20	49.11 ± 1.14 ^c^	45.23 ± 2.05 ^b^	43.78 ± 3.15 ^b^	29.24 ± 2.79 ^a^	48.79 ± 1.18 ^c^	47.17 ± 1.22 ^bc^

ASC (L-ascorbic acid); BHT (2,6-di-tert-butyl-4-methyl phenol). Data are expressed as the mean ± SEM (*n* = 3); values with different superscript letters across the rows are significant at *p* < 0.05.

**Table 4:** DPPH radical-scavenging activity of *C. limon* essential oils.

	Mean Percentage Inhibition of the DPPH Radical (%)				IC_50_ ± SEM (µg mL^−1^)
*C. limon* Oil	20 µg mL^−1^	40 µg mL^−1^	80 µg mL^−1^	160 µg mL^−1^	
Fresh leaf	43.44 ± 1.74 ^b^	54.89 ± 0.43 ^a^	62.93 ± 0.28 ^b^	73.75 ± 0.15 ^a^	34.54 ± 3.01 ^e^
Dry leaf	50.78 ± 0.69 ^c^	56.95 ± 0.87 ^b^	66.88 ± 0.56 ^d^	84.01 ± 0.45 ^b^	12.59 ± 1.48 ^c^
Fresh peel	45.32 ± 2.56 ^b^	55.92 ± 0.80 ^ab^	64.27 ± 0.21 ^c^	75.82 ± 0.61 ^a^	27.15 ± 2.72 ^d^
Dry peel	52.41 ± 0.26 ^d^	58.70 ± 0.62 ^c^	67.00 ± 0.02 ^e^	87.68 ± 0.01 ^c^	8.79 ± 1.30 ^b^
ASC	54.25 ± 3.55 ^cd^	61.09 ± 1.17 ^d^	70.08 ± 0.17 ^f^	92.81 ± 0.95 ^d^	3.01 ± 0.23 ^a^
BHT	36.59 ± 2.85 ^a^	55.82 ± 5.51 ^abcd^	60.67 ± 0.32 ^a^	73.53 ± 4.79 ^a^	27.58 ± 3.16 ^d^

Data are expressed as the mean ± SEM (*n* = 3) concentration that caused 50% inhibition of the DPPH radical (IC_50_); values with different letters in superscript (along the columns) are significant at *p* < 0.05. ASC (L-ascorbic acid); BHT (2,6-di-tert-butyl-4-methyl phenol).

**Table 5:** Repellent activity of *C. limon* EOs against *S. zeamais*.

*C. limon* Oil	Repellent Activity (%)				Statistical Analysis	
	10 µL	20 µL	30 µL	40 µL	F_3,56_	*p*-Value
Dry peel	52.00 ± 5.09	69.33 ± 5.47	57.33 ± 5.81	82.67 ± 4.73	6.6455	<0.001
Dry leaf	73.33 ± 6.95	72.00 ± 5.45	82.67 ± 4.73	78.33 ± 2.82	1.4180	>0.05

**Table 6:** Fumigant toxicity of *C. limon* peel EOs against *S. zeamais*.

*C. limon* Essential Oil	Exposure Time (h)	No. of Insects Tested	LC_50_ (95% CI) (µL mL^−1^ air)	Slope ± SEM	χ^2^ (df)	*p*-Value
Dry peel	48	150	0.34 ^b^	1.35 ± 0.55	17.92 (10)	>0.05
	72	150	0.28 (0.13–49.30)	1.20 ± 0.50	13.62 (10)	>0.05
	96	150	0.08 (0.06–0.16)	2.15 ± 0.49	4.21 (10)	>0.05
Dry leaf	48	150	0.17 (0.12–0.48)	3.08 ± 0.96	5.45 (10)	>0.05
	72	150	0.09 (0.07–0.11)	3.11 ± 0.61	7.87 (10)	>0.05
	96	150	0.06 (0.05–0.07)	2.54 ± 0.49	5.58 (10)	>0.05

LC_50_ values represent the concentrations causing 50% mortality; log concentrations in µL mL^−1^ air were used to calculate the LC_50_ values; *n* = 3 replicates of 10 maize weevils. Slope is not significantly different from zero ^b^.

**Table 7:** Contact toxicity of *C. limon* dry peel and leaf EOs against *S. zeamais*.

*C. limon* Essential Oil	Exposure Period (h)	Number of Weevils Tested	LC_50_ (95% CI) (µL g^−1^)	Slope ± SE	χ^2^ (df)	*p*-Value
Dry peel	24	300	2.02 (1.76–2.27)	4.07 ± 0.46	8.29 (10)	>0.05
	48	300	1.66 (1.41–1.89)	3.82 ± 0.49	10.80 (10)	>0.05
	72	300	1.39 (0.95–1.74)	3.34 ± 0.47	15.33 (10)	>0.05
Dry leaf	24	300	4.09 (3.01–5.61)	3.94 ± 0.44	42.02 (10)	<0.001
	48	300	3.19 (2.44–4.11)	2.91 ± 0.34	21.84 (10)	<0.05
	72	300	2.39(1.76–3.02)	2.92 ± 0.35	20.41(10)	<0.05

LC_50_ values are considered significantly different( when the 95% confidence intervals (CIs) do not overlap. Log concentrations in µL g^−1^ air were used to calculate the LC_50_ values; *n* = 3 replicates of 20 maize weevils.

**Table 8:** Simulated agricultural and socio-economic benefits of using *C. limon* peel waste generated in South Africa.

Simulated Agricultural and Socio-Economic Benefits of *C. limon* (Lemon) Peel Waste	Values
Total amount of lemons produced (2023)	653,000 MT
Total amount of lemons imported	3000 MT
Total amount of lemons exported (2023)	573,000 MT
Total amount of lemons consumed/wasted within South Africa (2023)	83,000 MT
Average weight (wet) of fruit peel per lemon	39.70 g
Average dehydrated (dry) of fruit peel per lemon	9.19 g
Total available dehydrated lemon peel within South Africa (2023)	0.762 MT
Total maize used for animal feed (2023) in South Africa	6.650 MT
Cost of lemon peel as an alternative energy-producing ingredient in animal feed (at the current maize market price of ZAR 3754/ton)	ZAR 2,869,548,000:00
Cost of maize used in animal feed (2024) in South Africa (January 2024 market price/ton)	ZAR 24,964,100,000:00

MT—million tons, g—grams.

## Data Availability

Data are contained within the article.
